# Development of a Multiplex RT–qPCR Method for the Identification and Lineage Typing of Porcine Reproductive and Respiratory Syndrome Virus

**DOI:** 10.3390/ijms252313203

**Published:** 2024-12-08

**Authors:** Chunhao Tao, Xizhou Zhu, Ying Huang, Weifeng Yuan, Zhen Wang, Hongfei Zhu, Hong Jia

**Affiliations:** 1 Institute of Animal Sciences, Chinese Academy of Agricultural Sciences, Beijing 100193, China; chunhao_tao@163.com (C.T.);; 2 Bioproducts Engineering Center, Chinese Academy of Agricultural Sciences, Beijing 100193, China; zhu.xizhou@hotmail.com; 3 Institute of Comparative Medicine, College of Veterinary Medicine, Yangzhou University, Yangzhou 225009, China; hy811cysbm@163.com

**Keywords:** PRRSV, identification, lineage typing, multiplex, diagnosis

## Abstract

Porcine reproductive and respiratory syndrome virus (PRRSV) is the pathogen that causes porcine reproductive and respiratory syndrome (PRRS), leading to abortion of sows and the manifestation of respiratory diseases in piglets. PRRSV strains are categorized into two distinct genotypes: PRRSV–1 and PRRSV–2. PRRSV–2 can be further classified into several lineages, including sub–lineage 1.8 (NADC30–like), sub–lineage 1.5 (NADC34–like), lineage 8 (HP–PRRSV–like), lineage 5 (VR–2332–like), and lineage 3 (QYYZ–like), all of which are prevalent in China. In order to identify PRRSV–1 and PRRSV–2, two primer–probe combinations were designed, targeting the M gene. In order to further differentiate the five lineages of PRRSV–2, another five primer–probe combinations were designed, targeting the Nsp2 gene. A TaqMan–based multiplex RT–qPCR assay was subsequently developed, integrating the aforementioned seven sets into two primer pools. Following the optimization of primer concentration and annealing temperature, a comprehensive evaluation was conducted to assess the assay’s amplification efficiency, specificity, repeatability, and sensitivity. The developed multiplex RT–qPCR method exhibited excellent repeatability, with coefficients of variation (CVs) less than 2.12%. The detection limits for all seven targets were found to be less than 5 copies/μL. Ultimately, the method was utilized for the detection of a total of 1009 clinical samples, with a PRRSV–positive rate of 7.63% (77/1009). Specifically, the reference method was utilized to further confirm the status of the 77 PRRSV–positive samples and another 27 samples suspected of PRRSV infection. The sensitivity of the method was 97.40% (75/77), and the specificity was 96.30% (26/27), resulting in an overall coincidence rate of 97.12% (101/104). All the PRRSV–positive samples were typed as NADC30–like strains, and the accuracy of this typing was further confirmed by Sanger sequencing. In conclusion, A one–step multiplex RT–qPCR method was successfully constructed, evaluated, and applied to detect clinical samples. The assay provides an easy–to–operate, time–saving, and highly efficient way for the quick identification of PRRSV and simultaneous detection of five PRRSV–2 lineages prevalent in China. The method could offer guidance for PRRSV prevention and control measures.

## 1. Introduction

Porcine reproductive and respiratory syndrome (PRRS), caused by porcine reproductive and respiratory syndrome virus (PRRSV), poses a significant threat as a highly contagious disease to the pig farming industry. PRRS possesses extremely strong transmission capabilities and has now swept across the globe, wreaking havoc, particularly in Europe, America, and Asia [1,2]. Infection with PRRSV can result in reproductive failure in pregnant sows and pneumonia in piglets [3]. PRRSV primarily targets porcine alveolar macrophages, eliciting a spectrum of effects that encompass immunosuppression, persistent infection, secondary infection, and often co–infection with other pathogens [3,4]. Therefore, the mortality rate of pigs due to PRRSV infection stands prominently among all porcine diseases [5]. PRRSV is an enveloped virus belonging to the Arteriviridae family with a positive–sense (+) RNA genome [6]. The PRRSV genome consists of 12 open reading frames, coding for 16 nonstructural proteins and 8 structural proteins [7].

As an RNA virus, PRRSV has undergone significant evolutionary diversification, giving rise to numerous distinct lineages. All PRRSV strains can be categorized into two distinct genotypes. PRRSV–1 and PRRSV–2 are distinguished by the originally isolated strains, designated Lelystad Virus (the European prototypic strain) and ATCC VR–2332 (the North American prototypic strain), respectively [8]. PRRSV–1 and PRRSV–2 exhibit only 50–70% nucleotide sequence identity and have been classified as two distinct species since 2021 based on antigenicity [9]. PRRSV–1 can be divided into three or four types, with only subtype Ⅰ (Global) being prevalent in China [10]. PRRSV–2 has been classified into nine lineages based on the ORF5 gene sequence, with five lineages (lineage 1, 3, 5, 8, and 9) being prevalent in China [11,12,13,14]. The predominant PRRSV–2 strains in China are sub–lineage 1.8 (NADC30–like) strains, sub–lineage 1.5 (NADC34–like) strains, lineage 8 (HP–PRRSV–like) strains, lineage 5 (VR–2332–like) strains, and lineage 3 (QYYZ–like) strains [13,14,15,16]. The phenomenon of deletions in the Nsp2 protein is commonly observed in various PRRSV lineages. For instance, HP–PRRSV–like strains show a non–consecutive removal of 30 amino acids (aa), whereas NADC30–like strains exhibit a similar fragmented deletion encompassing 131 aa. Additionally, NADC34–like strains undergo a continuous truncation of 100 aa [17].

A variety of techniques, including serological methods and molecular biology methods, can be utilized to identify PRRSV [18]. To differentiate various types of PRRSV, the TaqMan–based reverse transcription real–time quantitative PCR (RT–qPCR) is an optimal choice, as it allows for simultaneous detection of four targets within a “close–tube” system, while also offering notable time–saving capabilities and superior sensitivity [19]. The M protein gene (ORF6) and N protein gene (ORF7) exhibit a higher degree of conservation within the complete genome of PRRSV, frequently serving as targets for the identification of PRRSV–1 and PRRSV–2 [20,21,22]. Given the distinctive deletion pattern mentioned before, the highly variable Nsp2 protein has been employed as a frequently utilized target for further lineage typing of PRRSV–2 [21,23,24,25].

PRRSV strains are categorized into diverse lineages and exhibit a wide spectrum of pathogenicity. Furthermore, it has been demonstrated that PRRSV vaccines provide only limited cross–protection against heterogeneous strains [26]. It is therefore imperative that the prompt and precise typing of PRRSV strains is established as a crucial reference for the mitigation and management of PRRS. However, the methodologies presently established are limited to genotyping just two to three predominant subtypes of PRRSV–2 strains [20,21,27]. In this study, a TaqMan–based multiplex RT–qPCR method for the rapid identification and typing of PRRSV strains was developed. This method is capable of distinguishing between PRRSV–1 and PRRSV–2, and further typing five PRRSV–2 lineages: NADC30–like strains, NADC34–like strains, HP–PRRSV–like strains, VR–2332–like strains, and QYYZ–like strains. This method has significant potential as an effective diagnostic and monitoring tool for PRRSV, facilitating timely intervention and management strategies.

## 2. Results

### 2.1. Establishment of Multiplex RT–qPCR Method

#### 2.1.1. Validation of Single Primer–Probe Combinations

To guarantee the accuracy and reliability of the primers and probes, a single combination was incorporated into the RT–qPCR reaction system, utilizing the corresponding plasmid containing the target gene as a template (10^4^ copies/μL). As illustrated in Supplementary Table S1, the amplification curve and Ct value could only be discerned when the corresponding plasmid containing the target gene was employed, thereby attesting to the efficacy and precision of the primers and probes. 

#### 2.1.2. Validation of Two Primer Pools

Given the restricted number of detection targets in real-time PCR, we allocated four primer–probes to one primer pool, designated as Primer Pool A (P.A), and the remaining three primer–probes to another primer pool, designated as Primer Pool B (P.B). Similarly, we evaluated the efficacy of the two primer pools. The results also indicated satisfactory efficacy for both primer pools (Table 1).

#### 2.1.3. Optimization of Concentration of Primers and Probes in Two Primer Pools

**Figure 1:**
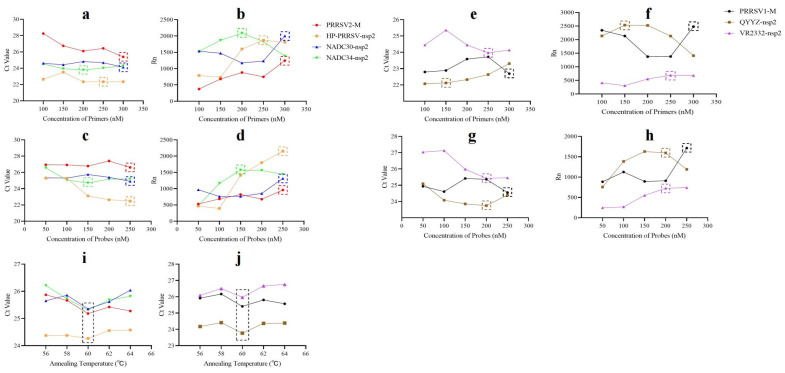
Optimization of reaction system. (**a**) The mean Ct value for each concentration of primers in P.A. (**b**) The mean Rn value for each concentration of primers in P.A. (**c**) The mean Ct value for each concentration of probes in P.A. (**d**) The mean Rn value for each concentration of probes in P.A. (**e**) The mean Ct value for each concentration of primers in P.B. (**f**) The mean Rn value for each concentration of primers in P.B. (**g**) The mean Ct value for each concentration of probes in P.B. (**h**) The mean Rn value for each concentration of probes in P.B. (**i**) The mean Ct value for each annealing temperature of P.A. (**j**) The mean Ct value for each annealing temperature of P.B. The dotted boxes indicate the optimal concentration or annealing temperature selected. Each test was conducted with three replicates.

In order to identify the most appropriate concentration of a pair of primers within P.A, the concentrations of the corresponding probe and all other primer–probes were fixed, while the concentration of the primer of interest was varied from 100 nmol/L to 300 nmol/L. The mean Ct values for each reaction are displayed in Figure 1a, and the absolute fluorescence signal (Rn) value is presented in Figure 1b. The concentration that resulted in a lower Ct value was selected as the optimal concentration. However, when the Ct values were equally low, the concentration with a higher Rn value was selected. Similarly, concentrations of probes in P.A (Figure 1c,d), primers in P.B (Figure 1e,f), and probes in P.B (Figure 1g,h) were optimized. Finally, the best concentrations for all primers and probes were selected (Figure 1).

#### 2.1.4. Optimal Annealing Temperature

Following the identification of the optimal reaction system, an examination of reaction conditions was conducted using a range of annealing temperatures ranging from 56 °C to 64 °C. The temperature that resulted in a lower Ct value was selected as the optimal annealing temperature. The results demonstrated that the optimal annealing temperature for both primer pools was 60 °C (Figure 1i,j).

### 2.2. Construction of Standard Curves

**Figure 2:**
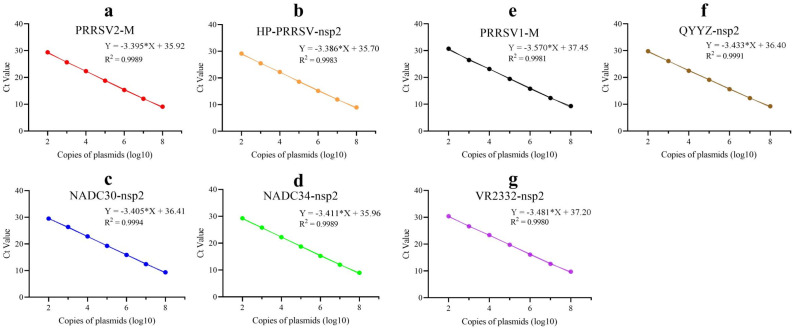
Constructions of standard curves. (**a**–**d**) Standard curves of four primer–probes in P.A. (**e**–**g**) Standard curves of three primer–probes in P.B. Each test was conducted with three replicates. The asterisk (*) represents the multiplication sign in linear equations.

To ascertain the efficacy of the amplification process, standard plasmids were identified using the optimized methodology, employing a series of dilutions spanning a range from 10^8^ copies/μL to 10^2^ copies/μL. The standard curves exhibited excellent linear relationships, with R^2^ values exceeding 0.99 for all four primer–probes in P.A (Figure 2a–d) and all three primer–probes in P.B (Figure 2e–g). The amplification curves are presented in Supplementary Figure S1. The amplification efficiency of each primer–probe was calculated based on the slope of the corresponding standard curves, with the following values obtained: 97.0% for PRRSV2–M, 97.4% for HP–PRRSV–nsp2, 96.6% for NADC30–nsp2, 96.4% for NADC34–nsp2 in P.A, and 90.6% for PRRSV1–M, 95.6% for QYYZ–nsp2, 93.8% for VR2332–nsp2 in P.B. The results above indicate that both primer pools have great amplification efficiency.

### 2.3. Specificity Tests

**Figure 3:**
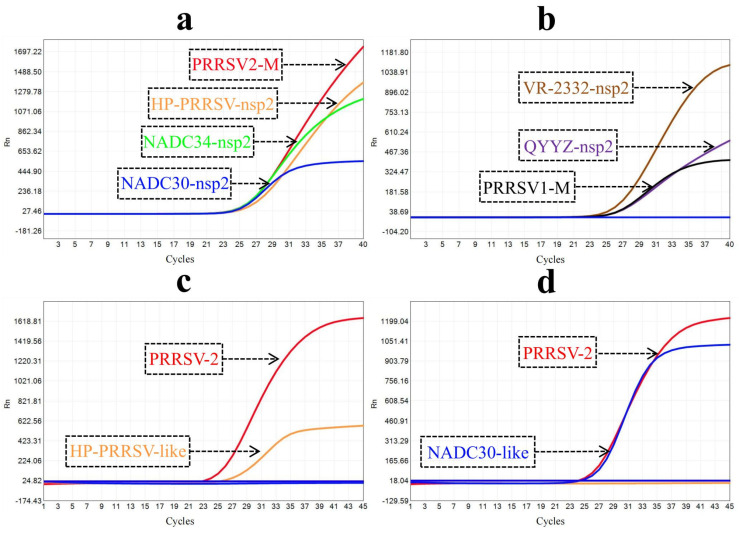
Amplification curves of specificity tests. (**a**) Amplification curves of four mixed plasmids corresponding to P.A. (**b**) Amplification curves of three mixed plasmids corresponding to P.B. The concentration of each plasmid is 10^4^ copies/μL. (**c**) Amplification curves of the HP–PRRSV–like (FZ16A) strain detected using P.A. (**d**) Amplification curves of the NADC30–like (GXNN202004a) strain detected using P.A. The red, orange, blue, green, black, brown, and purple curves represent the genotypes or lineages of PRRSV–2, HP–PRRSV–like, NADC30–like, NADC34–like, PRRSV–1, VR–2332–like, and QYYZ–like, respectively. Amplification curves for four mixed plasmids detected with P.B, three mixed plasmids detected with P.A, and two PRRSV strains detected with P.B are not shown as no amplification curves were detected.

The established method demonstrated the capacity to detect all corresponding plasmids containing target genes while exhibiting no discernible results when utilizing other non–corresponding plasmids (Figure 3a,b). Furthermore, the method demonstrated the capacity to accurately type two PRRSV–2 strains (Figure 3c,d). The nucleic acids of African swine fever virus (ASFV), pseudorabies virus (PRV), porcine epidemic diarrhea virus (PEDV), porcine circovirus type 2 (PCV2), and classical swine fever virus (CSFV) showed no amplification curves when employed as templates in our detection method. These findings collectively demonstrate the high specificity of our approach.

### 2.4. Repeatability Tests

To assess the repeatability of the method, high and low copy numbers (10^5^ and 10^2^ copies/μL, respectively) of plasmids were employed. The Ct values were observed to be between 18 and 20 when amplifying high copies of plasmids, with coefficients of variation (CVs) ranging between 0.40% and 1.15% (Table 2). Meanwhile, the Ct values were observed to be between 29 and 31 when amplifying low copies of plasmids, with CVs ranging between 0.99% and 2.12% (Table 2). These results demonstrate that the method exhibits excellent repeatability. The amplification curves for the repeatability tests are available in Supplementary Figure S2.

### 2.5. Sensitivity Tests

In order to verify the sensitivity of the established method, standard plasmids were diluted to concentrations of 10 copies/μL, 5 copies/μL, 1 copy/μL, and 0.5 copies/μL and then subjected to detection procedures. The results demonstrate that when the plasmid concentration was 5 copies/μL, at least 95% of 20 detection repeats were successfully identified (Table 3), indicating that the method is capable of detecting down to 5 copies/μL for all seven targets. The amplification curves of the sensitivity tests are available in Supplementary Figure S3.

### 2.6. Detection of Clinical Samples

A total of 1009 samples were collected from three farms and subjected to detection using the established multiplex RT–qPCR method. Of the samples tested, 7.63% (77/1009) were positive for PRRSV, while 92.37% (932/1009) were negative. Notably, among the 932 negative samples, 27 were judged to be PRRSV–suspect due to their Ct values exceeding 39, despite being initially classified as negative. All the PRRSV–positive samples were of the PRRSV–2 genotype and were further typed as the NADC30–like lineage. To validate the efficacy of our RT–qPCR method, the national reference method was employed in parallel to ascertain the status of the 104 PRRSV–suspect samples (77 positive and 27 suspected based on our method). Finally, the results demonstrate that the sensitivity of the developed RT–qPCR method is 97.40% (75/77), and the specificity is 96.30% (26/27), with an overall coincidence rate of 97.12% (101/104) (Table 4). To corroborate the precision of the lineage classification with our RT–qPCR method, eight selected samples were subjected to PCR detection and sequencing. The findings revealed that the Nsp2 gene sequence of these samples was identical and aligned with the NADC30–like lineage, as delineated by the patterns of amino acid deletions. 

## 3. Discussion

PRRS has been a significant concern in the pig farming industry for nearly three decades. Currently, it is the second most problematic disease after African swine fever, particularly in China. PRRSV has undergone a process of evolution, resulting in the emergence of diverse lineages. The recombination of genetic material between or within lineages is a highly prevalent phenomenon [28,29,30]. PRRSV of different lineages typically exhibits varying degrees of pathogenicity [29,30,31]. The efficacy of vaccinations utilizing vaccines derived from disparate lineages has been demonstrated to be limited [32]. It is therefore of great importance to accurately identify the different genotypes and lineages of PRRSV, as this facilitates both the selection of appropriate vaccines and the analysis of PRRS epidemiological trends associated with PRRSV. In this article, we present a multiplex RT–qPCR method that can identify two genotypes of PRRSV (PRRSV–1 and PRRSV–2) and further type five lineages of PRRSV–2 (HP–PRRSV–like, NDC30–like, NADC34–like, QYYZ–like, and VR–2332–like). Our methodology encompasses nearly all subtypes of PRRSV prevalent in China. Although it does not cover some subtypes prevalent in other countries [33], this method remains applicable for the identification of PRRSV in those nations and for the further subtyping of common subtypes, such as the NADC30–like strains and VR–2332–like strains [34,35].

PRRSV–2 can be classified into nine lineages based on the ORF5 gene [11]. However, it is challenging to identify specific regions between different lineages due to the limited length of the ORF5 gene, which is approximately 600 nucleotides, and the high frequency of mutations within this gene [35]. The highly variable Nsp2 gene comprises over 3000 nucleotides and exhibits unique deletion patterns among different lineages, rendering it a suitable substitute for the ORF5 gene in lineage typing [17,18]. A variety of methods have been devised for the identification of the HP–PRRSV–like, NADC30–like, and VR–2332–like strains [20,21,24]. Accordingly, five primer–probe combinations were devised targeting the Nsp2 gene for the purpose of typing the five lineages of PRRSV–2 strains that are prevalent in China. It is important to note that phylogenetic analysis based on distinct genes may not be entirely comparable, particularly in the context of recombinant events involving the ORF5 and Nsp2 genes, which are common occurrences. Three recombinant strains were assigned to the NADC34–like lineage, yet they were classified as NADC30–like strains according to the whole genome and Nsp2 evolutionary trees [30]. It should be noted that occurrences of lineage typing relying on the RT–qPCR method can merely serve as a reference for rapidly identifying the PRRSV lineages based on the corresponding gene. Whole–genome sequencing and analysis continue to play a vital role in the exploration of the evolutionary patterns of the virus [36].

In the formation of primer pools, PRRSV–1 and PRRSV–2 genotyping primer–probes were assigned to distinct pools. The rationale behind this approach is twofold. Firstly, PRRSV–2 lineages such as NADC30–like, NADC34–like, and HP–PRRSV–like are predominant in China. Secondly, it is possible that PRRSV–1 primers may exhibit cross–reactivity with the PRRSV–2 probe, which is itself nonspecific. Nevertheless, despite these considerations, the composition of the primer pools has no influence on the detection results.

The previously developed quadruplex RT–qPCR assays generally require the completion of a reverse transcription step before carrying out the qPCR. This additional step increases the cost in terms of time [20,21]. In contrast, the time required for our method is reduced, as the one–step RT–qPCR kit allows for the direct detection of extracted RNA. Furthermore, the high level of amplification efficiency (96.4–97.4% for P.A, 90.6–95.6% for P.B), repeatability (CVs less than 2.12% for 10^2^ copies/μL plasmids), and sensitivity (limit of detection less than 5 copies/μL) indicates that the method has significant potential for application in PRRSV diagnosis. Additionally, the superior performance and broad−spectrum genotyping capability of this method can provide rapid reference for the utilization of vaccines in clinical settings, thereby aiding in the prevention of PRRS.

The developed RT−qPCR method was employed for the screening of 1009 clinical samples, with all PRRSV−positive samples subsequently confirmed as belonging to the PRRSV−2 strain. For purposes of comparison, the widely adopted national reference RT−qPCR method, tailored for the identification of the PRRSV−2 strain, was also applied to these samples [22]. The results demonstrated a high degree of concordance between our developed method and the national reference method. To further validate the accuracy of lineage typing, we initially employed a comparable RT−qPCR method capable of detecting VR−2332−like, HP−PRRSV−like, and NADC30−like PRRSV strains [25]. However, this alternative method yielded negative results for NADC30−like PRRSV in all clinical samples, potentially due to the high variability of the Nsp2 gene in PRRSV. To address this challenge, eight samples with manageable Ct values (indicating sufficient virus copies for PCR amplification) were selected and subjected to PCR assays and Sanger sequencing for lineage verification. It is noteworthy that all eight samples were confirmed as NADC30−like strains, thereby confirming the accuracy and broad applicability of our established method. The identical Nsp2 gene sequences among these eight samples may be attributed to the fact that they were collected from the same farm and likely originated from the same strain prevalent among the pigs. In conclusion, the comprehensive results demonstrate that our developed method can precisely identify PRRSV strains and effectively type at least the NADC30−like lineage.

The developed RT−qPCR method is capable of identifying both PRRSV−1 and PRRSV−2 strains. Furthermore, the method is capable of simultaneously differentiating between five distinct lineages of PRRSV-2 strains that are prevalent in China, and it is capable of detecting instances of coinfection with PRRSV. Previous methods have primarily focused on two or three PRRSV lineages, including NADC30−like strains, HP−PRRSV−like strains, and VR−2332−like strains [20,21,23,24,25]. To date, no reported method has been able to identify such a wide range of lineages simultaneously, thereby demonstrating the significant potential of our method as a powerful tool for the rapid diagnosis and typing of PRRSV. However, a limitation of this research is that the number of clinical samples available for verification of the method’s application across different lineages of PRRSV−positive strains was insufficient. Only NADC30−like strains were detected in our clinical sample tests. In the near future, we will endeavor to collect as many samples as possible to conduct more detailed clinical sample experiments. Furthermore, we intend to undertake substantial improvements to our method, focusing on two primary facets: one, by refining the strain genotyping identification process to encompass all prevalent virus strains worldwide, and two, by augmenting our detection capabilities, potentially through the incorporation of advanced technologies such as CRISPR−Cas and next−generation sequencing, to accurately detect PRRSV recombinant strains.

## 4. Materials and Methods

### 4.1. Virus, Nucleic Acids, and Clinical Samples

HP−PRRSV−like PRRSV strain (FZ16A, KY761966.1) was stored in our laboratory. NADC30−like PRRSV strain (GXNN202004a, MW531679.1) was generously provided by Zuzhang Wei from Guangxi University. The DNA/RNA of ASFV, PRV, PEDV, PCV2, and CSFV have been extracted and preserved in our laboratory. A total of 1009 clinical samples were collected from various places in China. Specifically, 710 samples were obtained from a farm located in Guangdong Province, encompassing swabs, sera, mandibular lymph nodes, mesenteric lymph nodes, inguinal lymph nodes, tonsils, lungs, and spleen. Swabs, sera, and tissue samples were randomly collected from different pigs in 16 barns. The remaining 299 samples, consisting of swabs and sera, were also randomly collected from different barns on two farms situated in Shandong Province. All the samples were subsequently transferred to the laboratory and stored at −80 °C.

### 4.2. Primers and Probes

A total of 269 PRRSV genome sequences were downloaded from GenBank, all of which were collected in China between 2018 and 2022. The complete sequences of the M gene and the Nsp2 gene from these sequences were extracted and aligned using the muscle algorithm in MEGA11 software. Two primer–probe combinations (PRRSV1−F/R/P, PRRSV2−F/R/P) were designed based on the conserved regions of the M gene with the objective of specifically detecting PRRSV−1 strains and PRRSV−2 strains, respectively. Additionally, five further primer–probe combinations (NADC30−F/R/P, NADC34−F/R/P, HP−PRRSV−F/R/P, VR−2332−F/R/P, QYYZ−F/R/P) were designed based on the conserved regions of Nsp2 gene, with the objective of specifically typing five lineages of PRRSV−2. The primers and probes are listed in Table 5. All oligonucleotides were synthesized by GenScript (Nanjing, China).

### 4.3. Extraction of Nucleic Acids and Construction of Standard Plasmids

Viral RNA was extracted from cultured virus strains and clinical samples, including sera and tissues, using the UE fluid DNA/RNA extraction kit (UElandy Inc., Cat. No. UE–MN–BF–VNA–250, Suzhou, China). The manufacturer’s instructions were followed. The extracted RNA was eluted with 50 μL of RNase–free ddH_2_O and stored at −80 °C for subsequent RT–qPCR analysis. The seven target genes (M or Nsp2) corresponding to a set of primers were synthesized by GenScript (Nanjing, China) and cloned into the pUC57 plasmid as standard plasmids. These standard plasmids were named PRRSV2–M, HP–PRRSV–nsp2, NADC30–nsp2, NADC34–nsp2, PRRSV1–M, QYYZ–nsp2, and VR–2332–nsp2. They were synthesized by GenScript (Nanjing, China) and dissolved in TE buffer (Sangon Biotech, Cat. No. B541019, Shanghai, China) at a concentration of 4 μg. The solutions were stored at −20 °C for further analysis.

### 4.4. Optimization of Reaction Condition

#### 4.4.1. Validation of Single Primer–Probe Combination and Two Primer Pools 

All seven standard plasmids were diluted to 10^4^ copies/μL and detected using the Hifair^®^ Ⅲ One Step RT–qPCR Probe Kit (Yeasen Biotechnology, Cat. No. 11145ES70, Shanghai, China). The reaction system and conditions were configured in accordance with the instructions provided by the manufacturer. To this mixture, 5 μL of plasmid templates were added. Subsequently, each distinct primer–probe combination was employed for the detection of all seven plasmids. Subsequently, four of the seven primer–probes (PRRSV2, HP–PRRSV, NADC30, NADC34) were combined to form P.A, while the remaining three (PRRSV1, QYYZ, VR–2332) were mixed to create P.B. The concentration of each primer–probe within these pools was standardized at 200 nmol/L and 100 nmol/L, respectively. Subsequently, 2 μL of each primer pool was introduced into the reaction system. Finally, both primer pools were employed to detect all seven plasmids, utilizing the Hifair^®^ Ⅲ One Step RT–qPCR Probe Kit. For the RT–qPCR runs, the SLAN–96S (manufactured by Shanghai Hongshi Medical Technology Co., Ltd., Shanghai, China) was used. During the RT–qPCR process, fluorescent signals from FAM, VIC, ROX, and Cy5 were captured. Following the completion of the reactions, the Ct values and Rn values of each channel were meticulously recorded.

#### 4.4.2. Optimization of Concentration of Primer–Probes in Two Primer Pools

In order to identify the most appropriate concentrations of a pair of primers (PRRSV2–F/R) in P.A, a series of tests was conducted, in which the concentrations of the primers were varied between 100 and 300 nmol/L while keeping the concentration of the corresponding probe (PRRSV2–P) and other primer–probe pairs were maintained at a fixed level. The reaction system is detailed in Table 6. The reaction procedure was carried out in accordance with the previously described methodology, and the Ct values and Rn values of each channel were recorded. Similarly, different concentrations (50–250 nmol/L) of one probe (PRRSV2–P) were tested, and the optimal concentrations of the other primer–probes were determined using the aforementioned method.

#### 4.4.3. Optimization of Annealing Temperature

The primer pools were reprepared using the optimal concentrations of primers and probes, as determined by preliminary trials. Subsequently, 10^5^ copies/μL of mixed standard plasmids were used as templates to prepare the reaction system. Following this, 2 μL of each primer pool was introduced into the reaction system. During the RT–qPCR, different annealing temperatures (56 °C, 58 °C, 60 °C, 62 °C, 64 °C) were tested, and the Ct values of each channel were recorded.

### 4.5. Standard Curve

The standard plasmids were serially diluted in a range from 10^8^ copies/μL to 10^2^ copies/μL. Each concentration of plasmids was tested using optimal reaction conditions, with the results recorded as Ct values of each channel after three repetitions. Following RT–qPCR, the amplification efficiency for each target was calculated using the slopes of the standard curves, in accordance with the methodology described in the book “Molecular Cloning: A Laboratory Manual” (Fourth Edition).

### 4.6. Specificity, Repeatability and Sensitivity

In addition to plasmids containing target genes, the nucleic acids of two PRRSV strains (HP–PRRSV FZ16A, NADC30–like GXNN202004a), as well as the nucleic acids of ASFV, PRV, PEDV, PCV2, and CSFV, were employed in specificity tests. Plasmids with 10^5^ and 10^2^ copies/μL were prepared and tested 20 times with two primer pools to ensure repeatability. Plasmids with 10, 5, 1, and 0.5 copies/μL were prepared and tested 20 times with two primer pools to determine the limit of detection for each primer–probe combination.

### 4.7. Detection of Clinical Sample

The sera and the swabs stored in physiological saline were directly employed for RNA extraction. All tissue samples were dissected into smaller fragments using sterile scissors. Subsequently, the tissue pieces were transferred to homogenization tubes, with 1 mL of physiological saline added as a buffer. Subsequently, all the tissue samples were homogenized using a homogenizer. The RNA of all samples was extracted in accordance with the aforementioned methodology. The eluted RNA was directly incorporated into the developed multiplex RT–qPCR reaction systems that had been developed for this purpose. Furthermore, a national reference qPCR method (GBT 35921–2018) was also employed as a comparison method to identify PRRSV [22]. In summary, the extracted RNA was reverse–transcribed into cDNA, which was then detected using the national reference method as previously described in the protocol. To confirm the lineage of the identified PRRSV strains, the Nsp2 genes of selected eight samples were amplified by PCR method and sequenced using Sanger sequencing. The primers employed for the amplification of the Nsp2 gene were 5′–CTGAVGGBAAYTGTGGTTGGCA–3′ (forward primer) and 5′–AARCCCCAATCACCCGGA–3′ (reverse primer). The lineage of the samples was ultimately determined based on the basis of the deletion pattern observed in the Nsp2 gene.

## 5. Conclusions

In conclusion, this study developed an efficient one–step multiplex RT–qPCR method for the identification and lineage typing of PRRSV. This method is time–saving and easy– to–operate. It is capable of typing nearly all prevalent PRRSV strains in China and demonstrates exceptional specificity, repeatability, and sensitivity. Overall, this method represents a highly valuable tool for contributing to the global surveillance of PRRS and informing the development of guidance for its prevention and management measures.

## 6. Patents

A patent application has been submitted for this research, with the following numbers: application (202410604903.4), online public examination (CN118308537A), and authorization (CN118308537B).

## Figures and Tables

**Table 1:** Results of validation of two primer pools.

		Primer Pool A				Primer Pool B		
		PRRSV2	HP-PRRSV	NADC30	NADC34	PRRSV1	QYYZ	VR-2332
Plasmid Templates	PRRSV2-M	26.1 ^1^	-	-	-	-	-	-
	HP-PRRSV-nsp2	-	24.26	-	-	-	-	-
	NADC30-nsp2	-	-	24.93	-	-	-	-
	NADC34-nsp2	-	-	-	24.36	-	-	-
	PRRSV1-M	-	-	-	-	25.17	-	-
	QYYZ-nsp2	-	-	-	-	-	24.72	-
	VR-2332-nsp2	-	-	-	-	-	-	26.06
	Mixed plasmids ^2^	30.14	28.67	28.35	28.11	29.18	29.42	30.46

^1^ The value refers to the Ct value of the RT–qPCR amplification curve. A minus sign indicates that there was no amplification curve detected for the reaction. ^2^ Mixed plasmids were obtained by mixing 7 plasmids containing the above targets in equal amounts.

**Table 2:** Results of repeatability tests.

Primer Pool	Plasmid Templates	Copies of Plasmids (Copies/μL)	Ct Value ^1^	CV (%)	CI ^2^
A	PRRSV2-M	10^5^	18.80 ± 0.12	0.66	[18.75, 18.85]
	HP-PRRSV-nsp2	10^5^	18.46 ± 0.18	0.96	[18.39, 18.53]
	NADC30-nsp2	10^5^	19.23 ± 0.08	0.40	[19.20, 19.26]
	NADC34-nsp2	10^5^	18.66 ± 0.14	0.74	[18.61, 18.72]
	PRRSV2-M	10^2^	30.23 ± 0.51	1.70	[30.02, 30.43]
	HP-PRRSV-nsp2	10^2^	29.93 ± 0.30	0.99	[29.81, 30.05]
	NADC30-nsp2	10^2^	30.73 ± 0.50	1.62	[30.53, 30.93]
	NADC34-nsp2	10^2^	30.15 ± 0.64	2.12	[29.90, 30.41]
B	PRRSV1-M	10^5^	19.33 ± 0.18	0.92	[19.25, 19.40]
	QYYZ-nsp2	10^5^	18.96 ± 0.08	0.40	[18.93, 18.99]
	VR2332-nsp2	10^5^	19.61 ± 0.23	1.15	[19.52, 19.70]
	PRRSV1-M	10^2^	30.92 ± 0.58	1.87	[30.69, 31.15]
	QYYZ-nsp2	10^2^	30.51 ± 0.56	1.85	[30.29, 30.74]
	VR2332-nsp2	10^2^	30.91 ± 0.55	1.79	[30.68, 31.13]

^1^ Each plasmid was tested 20 times. The mean Ct value and standard deviation were calculated and recorded in the table. ^2^ The 95% confidence interval has been calculated, with the number preceding the square brackets representing the lower limit of confidence and the number following it representing the upper limit of confidence.

**Table 3:** Results of sensitivity tests.

Primer Pool	Plasmid Templates	Positive Rates (%)			
		(10 Copies/μL)	(5 Copies/μL)	(1 Copies/μL)	(0.5 Copies/μL)
A	PRRSV2-M	100	100	10	15
	HP-PRRSV-nsp2	100	100	60	25
	NADC30-nsp2	100	95	30	0
	NADC34-nsp2	100	95	20	5
B	PRRSV1-M	100	95	20	0
	QYYZ-nsp2	100	100	40	0
	VR2332-nsp2	100	100	50	0

**Table 4:** Comparison between the developed RT–qPCR method and the national reference method.

Kappa Test		Developed Method		Total
		+	−	
Reference method	+	75	1	76
	−	2	26	28
Total		77	27	104

**Table 5:** Seven primer–probes designed for RT−qPCR.

Primers/Probes	Targets	Sequences (5′–3′)	Positions ^2^	Reference Strains
PRRSV1-F ^1^	PRRSV-1	CAGATGCAGATTGTGTTGCCT	14344–14364	OP566682.1
PRRSV1-R		CGGGCTTTCTCACAGCGTA	14450–14468	
PRRSV1-P		VIC-CCTGCAGCACTTTCTACG-MGB	14398–14415	
PRRSV2-F	PRRSV-2	TGCTAGGCCGCAAGTACAT	14592–14610	KY761966.1
PRRSV2-R		GGACGCCGGACGACAAA	14681–14697	
PRRSV2-P		Cy5-CTGGCCCCTGCCCACCAC-BHQ2	14612–14629	
HP-PRRSV-F	HP-PRRSV-like	CTATTACCCTGCACAAGGTGAC	2349–2370	KY761966.1
HP-PRRSV-R		GTGGACATGAGCCCATATTCTTC	2431–2453	
HP-PRRSV-P		ROX-TCTTCCAACTTAGAGAGTACG-MGB	2400–2420	
NADC30-F	NADC30-like	GATAGGGTGGAYATGCTAACCTG	2993–3015	OM293961.1
NADC30-R		GCATCATCACAAACCCGCAAG	3108–3128	
NADC30-P		FAM-ATGATACTCGAAACACC-MGB	3080–3096	
NADC34-F	NADC34-like	GTCGCTGGCAAGTACCTG	1089–1106	OL516348.1
NADC34-R		CTGTACAAYGATGGGTCCAC	1153–1172	
NADC34-P		VIC-TCGTGTCAGTCACTGC-MGB	1137–1152	
QYYZ-F	QYYZ-like	CACGCAGGAGTGGCTTTC	3311–3328	MN046242.1
QYYZ-R		CTCGAGAATCATCTTTGGGAGG	3416–3437	
QYYZ-P		ROX-CGCATGTGGGACAGGGTTGAC-BHQ2	3330–3350	
VR-2332-F	VR-2332-like	CACGCTCTTGTGCGACTG	1361–1378	MT746146.1
VR-2332-R		CGGGGAGTAGTGTTTGAGGT	1463–1482	
VR-2332-P		Cy5-CCGCGCTTTGTCCGTTCGTGA-BHQ2	1392–1412	

^1^ F stands for the forward primer, R stands for the reverse primer, and P stands for the probe. ^2^ The shown positions correspond to the genomes of the reference strains.

**Table 6:** Reaction system for optimization of concentration of primers in Primer Pool A.

Reaction Components	Volume (μL)
2 × Hifair^®^ Ⅲ P buffer	10
Hifair^®^ UH Ⅲ Enzymes	1
PRRSV2-F/R	0.2/0.3/0.4/0.5/0.6 ^2^
PRRSV2-P	0.4
HP-PRRSV-F/R/P ^1^	0.4
NADC30-F/R/P	0.4
NADC34-F/R/P	0.4
plasmid templates	3
nuclease-free water	up to 20 μL

^1^ F/R/P represent the forward primer, the reverse primer, and the probe, all of which were added into the reaction system. The concentrations of all primers and probes were 10 μmol/L. ^2^ Five different concentrations of the primers (PRRSV2–F/R) were tested.

## Data Availability

The data presented in this study are available in the Supplementary Materials here.

## Supplementary Materials

The following supporting information can be downloaded at: https://www.mdpi.com/article/10.3390/ijms252313203/s1.
