# Supplementary material for: Development of a Multiplex RT–qPCR Method for the Identification and Lineage Typing of Porcine Reproductive and Respiratory Syndrome Virus
Source: Int J Mol Sci. 2024 Dec 8;25(23):13203. doi: 10.3390/ijms252313203 (PMC11642648; doi:10.3390/ijms252313203)
Supplement: Supplementary file 1 [file ijms-25-13203-s001.zip › ijms-3362290-supplementary.pdf]

Table S1. Results of validation of single set of primers and probes.

		PRRSV2	HP-PRRSV	NADC30	NADC34	PRRSV1	QYYZ	VR-2332
Plasmid Templates	PRRSV2-M	25.12 [1]	-	-	-	-	-	-
	HP-PRRSV-nsp2	-	22.88	-	-	-	-	-
	NADC30-nsp2	-	-	23.4	-	-	-	-
	NADC34-nsp2	-	-	-	24.72	-	-	-
	PRRSV1-M	-	-	-	-	24.82	-	-
	QYYZ-nsp2	-	-	-	-	-	27.15	-
	VR-2332-nsp2	-	-	-	-	-	-	29.65

[1] The value refers to the Ct value of the RT-qPCR amplification curve. A minus sign indicates that there was no amplification curve detected for the reaction.

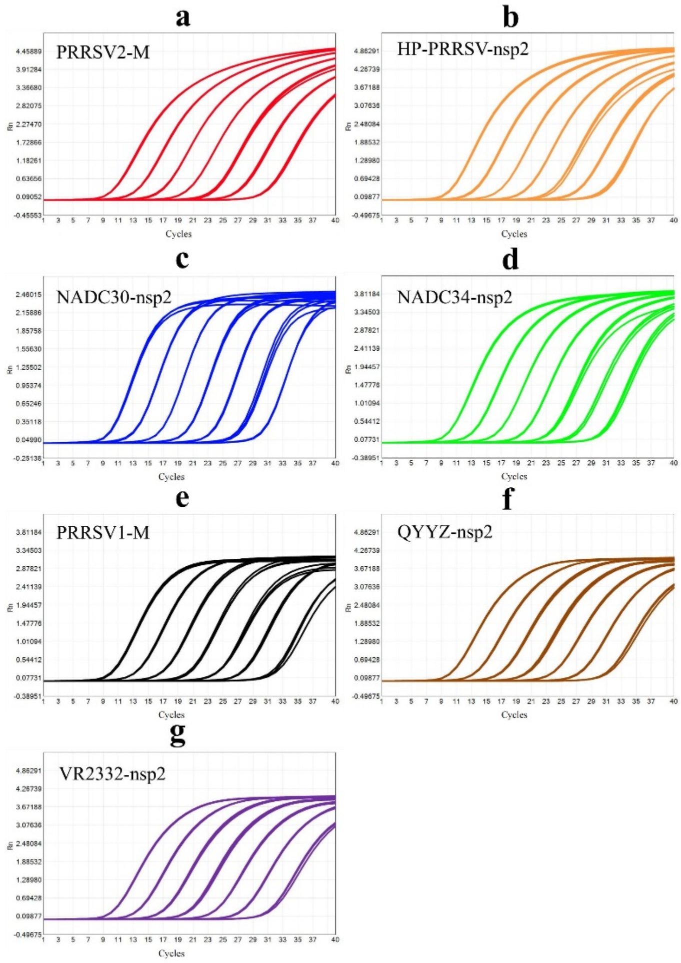

Figure S1. Amplification curves corresponding to standard curves. **(a-d)** Amplification curves of 4 primer-probes in primer pool A. **(e-g)** Amplification curves of 3 primer-probes in primer pool B. Each concentration of plasmids was detected three times.

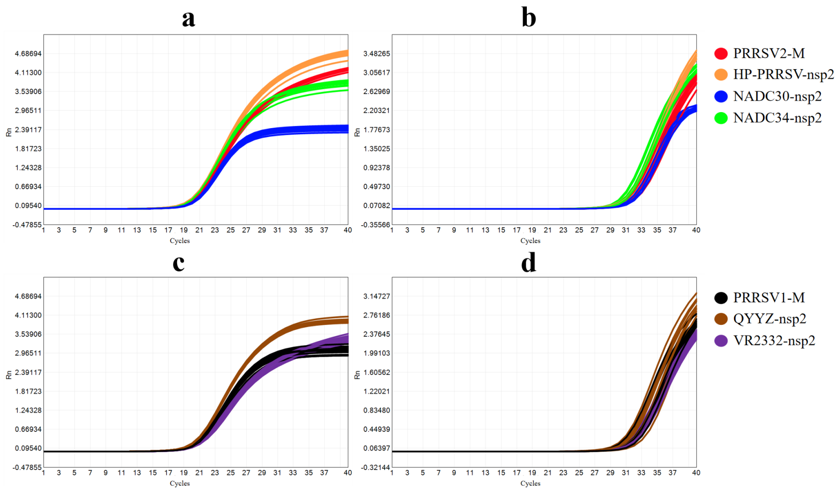

Figure S2. Amplification curves of repeatability tests. **(a)** Amplification curves of 4 primer-probes in primer pool A (plasmids 10^5 copies/μl). **(b)** Amplification curves of 4 primer-probes in primer pool A (plasmids 10^2 copies/μl). **(c)** Amplification curves of 3 primer-probes in primer pool B (plasmids 10^5 copies/μl). **(b)** Amplification curves of 3 primer-probes in primer pool B(plasmids 10^2 copies/μl). Each concentration of plasmids was detected twenty times.

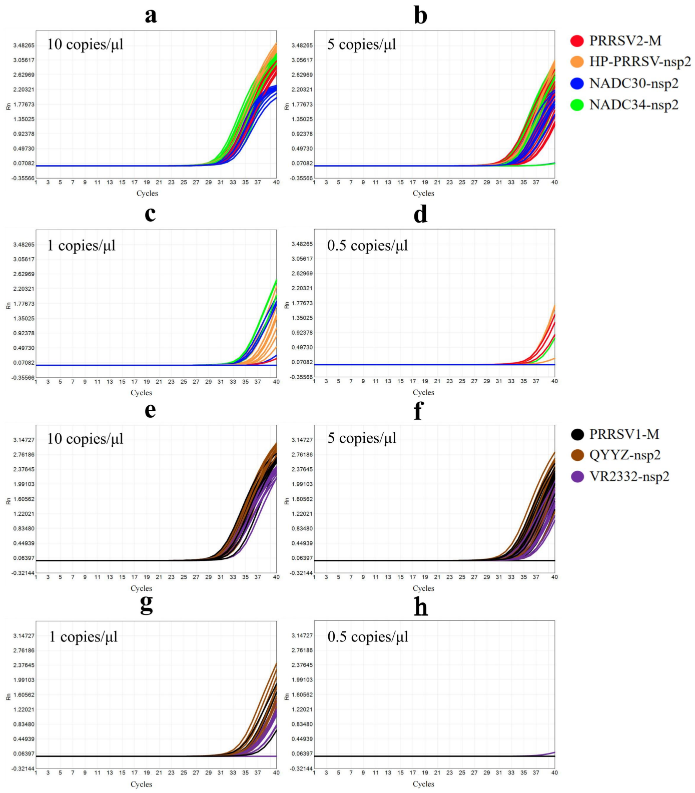

Figure S3. Amplification curves of sensitivity Tests. **(a-d)** Amplification curves of 4 primer-probes in primer pool A (plasmids 10^5 copies/μl to 0.5 copies/μl). **(e-h)** Amplification curves of 3 primer-probes in primer pool B (10^5 copies/μl to 0.5 copies/μl). Each concentration of plasmids was detected twenty times.
